# Anti-erythropoietin antibody levels and its association with anaemia in different strains of semi-immune mice infected with *Plasmodium berghei* ANKA

**DOI:** 10.1186/1475-2875-12-296

**Published:** 2013-08-27

**Authors:** Gideon Kofi Helegbe, Nguyen Tien Huy, Tetsuo Yanagi, Mohammed Nasir Shuaibu, Mihoko Kikuchi, Mahamoud Sama Cherif, Kenji Hirayama

**Affiliations:** 1 Department of Immunogenetics, Institute of Tropical Medicine (NEKKEN), Nagasaki University, 1-12-4 Sakamoto, Nagasaki 852-8523, Japan; 2 Department of Biochemistry and Molecular Medicine, School of Medicine and Health Sciences, University for Development Studies, Tamale, Ghana; 3 Animal Research Centre for Tropical Infections, Institute of Tropical Medicine (NEKKEN), Nagasaki University, 1-12-4 Sakamoto, Nagasaki 852-8523, Japan; 4 Global Center of Excellence, Institute of Tropical Medicine (NEKKEN), Nagasaki University, 1-12-4 Sakamoto, Nagasaki 852-8523, Japan

**Keywords:** Anti-EPO antibody, Erythropoietin, Malaria anaemia, *Plasmodium berghei* ANKA, Semi immune

## Abstract

**Background:**

Malaria anaemia is still a major public health problem and its pathogenesis still unclear. Interestingly, the progression of anaemia is at relatively low parasitaemia with some mortality in the semi-immune individuals in the endemic areas despite adequate erythropoietin (EPO) synthesis. A recent study has shown that treatment with exogenous anti-erythropoietin (anti-EPO) antibodies (Ab) of infected mice gives protection against malaria infection, suggesting an important role for anti-EPO Ab in malaria. The objective of the study was to evaluate anti-EPO antibody levels in anaemic condition of different strains of semi-immune mice with malaria.

**Methodology:**

Semi-immune status was attained in four mice strains (Balb/c, B6, CBA and NZW) by repeated infections with 10^4^*Plasmodium berghei* ANKA, and treatment with chloroquine/pyrimethamine. ELISA was used to measure anti-EPO Ab, transferrin and EPO while inflammatory cytokines measurement was done using bead-based multiplex assay kit.

**Results:**

The mean anti-EPO Ab levels in the mice strains [Optical Density (OD) values at 450 nm: Balb/c (2.1); B6 (1.3); CBA (1.4) and NZW (1.7)] differed (p = 0.045), and were significantly higher when compared with uninfected controls, p < 0.0001, and mean anti-EPO Ab levels in the mice strains at recovery [OD values at 450 nm: Balb/c (1.8); B6 (1.1); CBA (1.5) and NZW (1.0) also differed (p = 0.0004). Interestingly, EPO levels were significantly high in NZW and low in Balb/c mice (p < 0.05), with those of B6 and CBA of intermediary values. Again, NZW were highly parasitaemic (20.7%) and the other strains (Balb/c, B6 and CBA) ranged between 2.2-2.8% (p = 0.015). Anti-EPO Ab correlated positively with extent of Hb loss (r = 0.5861; p = 0.003). Correlation of anti-EPO antibody with EPO was significant only in Balb/c mice (r = −0.83; p = 0.01). Significant levels of IL6 and IFNγ (p < 0.0001), both known to be associated with erythropoiesis suppression were observed in the Balb/c. Transferrin was significantly lower in Balb/c (p < 0.0001) when compared with the other mice strains (B6, CBA and NZW).

**Conclusion:**

This is the first ever report in estimating endogenous anti-EPO antibodies in malaria anaemia. The data presented here suggest that anti-EPO Ab is produced at infection and is associated with Hb loss. Host factors appear to influence anti-EPO antibody levels in the different strains of mice.

## Background

Malarial infections result in a significant destruction of both infected red blood cells (iRBC) and uninfected red blood cells. It has been observed in chronic infected individuals or in the semi-immune, that the anaemia reported does not correlate with the level of parasitaemia [1,2]. Possible mechanisms that have been suggested are defective production of RBC or an excessive rate of red cells destruction or a combination of both [3,4]. However, studies have shown that erythropoietin (EPO) level is adequate in infected children and experimental models [5-7], and thus it is surprising that anaemia is not improved in that measure. Erythropoietin (EPO), a glycoprotein hormone, being a cytokine controls erythropoiesis or red blood cell production.

Studies in some auto-immune diseases and HIV patients revealed high level of anti-EPO auto-antibodies and its association with anaemia [8-10]. But this has not been studied in malaria anaemia cases. The use of EPO in therapy has been recommended to alleviate anaemia due to malarial infections [11] and also in cerebral malaria management [12]. The suggestion is that use of EPO will help a great deal in minimizing the risk of HIV/AIDS *via* blood transfusion when not screened properly. Meanwhile, EPO has been used successfully in a couple of diseases, treating anaemia in AIDS [13], in renal failure [14], as well as for limiting brain damage in experimental auto-immune encephalomyelitis [15], and also been proposed for treatment of haemoglobinopathies in which β-globin synthesis is affected [16,17]. These suggest an important role for EPO in therapy. However, neutralizing IgG antibodies to the protein component of exogenous recombinant EPO are found to cross-react with endogenous erythropoietin. Thus, the question that remains to be clarified is whether antibodies are produced against endogenous EPO and what the implications are during *Plasmodium* infections.

High level of anti-EPO auto-antibodies has been observed in some auto-immune diseases, but not reported in malaria anaemia (which has been thought to be auto-immune mediated). As a result anti-EPO antibodies may be implicated in malaria anaemia cases. Therefore, a study evaluating the levels of anti-EPO antibodies in malaria anaemia will be of interest to assess the benefits and/or predict (un)expected complications that may arise in the administration of exogenous EPO as therapeutic measure in malaria anaemia cases. Related to that, induction of antibodies against EPO molecule was observed in patients treated with recombinant human EPO which resulted in pure red cell aplasia [18]. Furthermore, the use of anti-EPO auto-antibodies as a therapy in murine malaria studies [19], is an indication of the important role it may play in severe malaria anaemia.

A previous study has shown that one mechanism of anaemia during Plasmodium infection was the destruction of uninfected red blood cells [2]. The current study aims to describe an alternative mechanism of anaemia by using sera raised from this previous study. Thus, the level of anti-EPO antibodies in malaria anaemia situation of different strains of semi-immune mice was evaluated. Furthermore, to investigate if host factors may play a role, different strains of semi-immune mice were used.

## Methods

### Mice, infection and generation of semi-immune status

**Figure 1:**
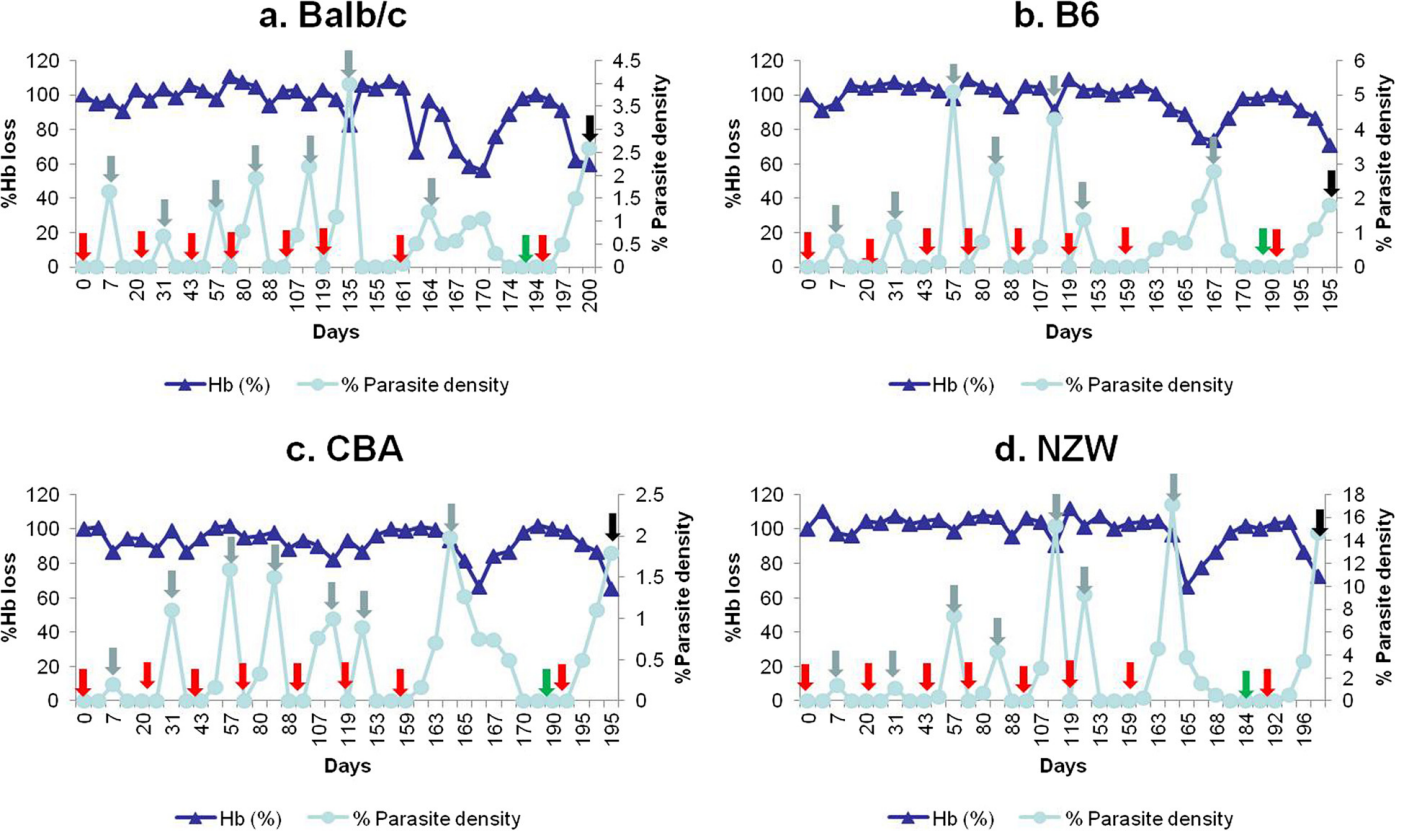
**Parasitaemia and haemoglobin profile in the mice strains during the seven cycles of infection and treatment.** The above data are representative data of one mouse per strain. Red arrows refer to infection with 10^4^*Plasmodium berghei* ANKA; blue arrows refer to treatment with chloroquine/pyrimethamine; green arrows refer to blood harvested at recovery after two weeks rest; black arrows refer to blood harvested during infection. Recovery refers to time at which mice has been rested for two weeks after treatment with chloroquine/pyrimethamine for 6 days or until parasitaemia is zero. Upper left graph **a** refers to profile for Balb/c; upper right graph **b** refers to profile for B6; lower left graph **c** refers to profile for CBA and lower right graph **d** refers to profile for NZW.

The procedure for this method has been reported elsewhere [2]. Briefly, four strains of mice BALB/c, C57BL/6 (B6), CBA and New Zealand White (NZW) aged 8 weeks supplied by SLC laboratories, Fukuoka, Japan, were injected intraperitoneally (i.p.) with 10^4^*Plasmodium berghei* ANKA (PbANKA)–iRBCs. Parasitaemia and reticulocyte levels were monitored every two days by Giemsa-stained thin blood film and are expressed as a percentage of more than 500 RBCs. Haemoglobin (Hb) was measured in a 96-well plate at 570 nm on Bio-Rad Model 3550 Micro plate Reader as previously described [2]. A recent study [20] has shown that estimates of polychromatophilic cells as done after Giemsa staining in this study are typically less than percentages of reticulocytes. However, Giemsa has been successfully used previously for reticulocyte staining [1,2]. Four microlitres of tail-vein blood was suspended in 1 mL Drabkin reagent (Sigma, St Louis, MO) and absorbance measured, and is expressed as a percentage of baseline levels. To generate semi-immune status, mice were treated at day 6 after infection with chloroquine/ (10 mg/kg intraperitoneally) and pyrimethamine (10 mg/kg intraperitoneally) daily for 6 days. During subsequent rounds of infection, mice were rested for two weeks before being rechallenged with 10^4^ PbANKA, then monitored and drug-cured prior to parasitemia reaching 5%. The mice strains were taken through 7 cycles of infection and treatment to generate the semi immune status, Figure 1. After the 7th cycle of infection and treatment (Figure 1), parasitaemia was checked and when it was negative (zero), which is the recovery stage, blood was collected from the tail, and serum harvested for analysis before the final cycle of infection without treatment. During the final cycle of infection (without treatment), mice were monitored every other day and, on days in which minimum Hb drop (i.e. maximum Hb reduction) was observed, blood was collected and sera were harvested for analysis, as described previously [2]. Sera were stored at −30°C until used. The sera used in this study were raised in previous study [2]. The parasitaemia and haematological profile of all the mice strains were, therefore, not reported here.

### Ethical statement

The study was conducted strictly according to the recommendation in the Fundamental Guidelines for Proper Conduct of Animal Experiment and Related Activities in Academic Research Institutions under the jurisdiction of the Ministry of Education, Culture, Sports, Science and Technology, Japan (Notice no. 71). All animal experiments were approved by the Nagasaki University, Board of Animal Research, according to Japanese Guideline for use of experimental animals (Permit Number 0811130716). All efforts were made to humanely minimize animal suffering.

### Screening of sera for anti-EPO antibodies by immunodot blot

Ultrabind US450 membrane (Gelman Sciences, Japan) was cut according to size and pre-wet with enough volume of tris-buffered saline (TBS). The membrane was placed unto the manifold and 300 μL TBS used to wash wells and membranes. Recombinant mouse EPO (R&D Systems, Japan, Catalogue Number 959-ME) was used as antigen to coat the membrane overnight at 4°C. The membrane in each well was blocked with 100 μL BlockAce (Catalogue No. UK-B25, Dainippon Pharmaceutical Co. Osaka, Japan) for an hour. Membranes in the wells were later washed once with 300 μL tris-buffered saline with tween (TTBS). Primary antibody (from infected sera and uninfected mice sera as negative control) were diluted at 1:10, 1:20 and incubated with the mouse EPO antigen for 3 hours at RT. The membranes in the wells were washed with 300 μL of TTBS twice before they were finally removed from the manifold and washed once in TTBS on a rotating machine. Secondary antibody of goat anti-mouse IgG-conjugated horse-radish peroxidase (HRP) at 1:2,000 dilution was incubated with the membrane for an hour at room temperature (RT) in the dark. Membrane was washed four times with TTBS and once with TBS to remove residual TTBS from membrane. Color development was done by addition of diaminobenzidine (DAB, at 5.0 mg/10 ml PBS + 5 uL H_2_0_2_) and incubated at RT for 10–15 min.

### Measurement of anti-EPO antibodies

This was done based on modified method according to Tzioufas *et al.*[9]. The recombinant mouse EPO (R&D Systems, Japan, Catologue Number 959-ME) was used as antigen. It was dissolved in phosphate buffered saline (PBS, pH 7.2), and coated in the polystyrene micro titer plate at 0.09 μg per well. After incubation overnight at 4°C, plates were washed with 0.1% Tween 20/PBS and blocking was done with Block Ace (Catalogue No. UK-B25, Dainippon Pharmaceutical Co. Osaka, Japan) for 1 hour. Diluted serum samples from the infected mice and uninfected mice (serving as negative control) all at 1:1,000 dilutions was added to the wells in duplicate and incubated for three hours at 37°C. After washing for five times with 0.1% Tween 20/PBS, 100 μL horse radish peroxidase (HRP)-conjugated goat anti-mouse IgG (Catalogue number 55563, MP Biomedicals, LLC 29525 Fountain Pkwy Solon, OH 44139, USA) at 1:2,500 was added to the wells in the 96-well ELISA plates and incubated for an hour at RT. Washing was done five times with 0.1% Tween 20/PBS and reaction visualized by the addition of 100 μL stabilized chromogen (Part/Lot number SS01/302008, Biosource, Carmarillo, CA, USA). Reaction was stopped 20 minutes later by the addition of 50 μL 1 N H_2_SO_4_ and absorbance was measured in the ELISA reader machine at 450 nm.

### Measurement of EPO levels

Measurement of mouse EPO was done according to manufacturer’s instructions of the Quantikine ELISA kit for mouse/rat EPO Immunoassay (R and D Systems, Japan, Catalogue number MEP00). Briefly 50 μL of standards, control and samples were pipette into the wells of a monoclonal antibody specific for mouse/rat EPO pre-coated micro- plate, which already has 50 μL of the assay diluents added. This was incubated at RT for 2 h on a shaker. The wells were washed five times and 100 μL of the conjugate added, then incubated at RT for 2 h on the shaker. Again washing was done five times with 100 μL substrate solution added to each well and incubated for 30 min on the bench top in the dark. After 100 μL stop solution was added to each and OD read at 450 nm.

### Measurement of inflammatory cytokines

Cytokines measurement was done using Procarta Mouse Cytokine Assay Kit plex according to manufacturer’s instructions (Luminex, Affymetrix). Briefly, filter plate was wetted with reading buffer and incubated at room temperature for 5 min, then filtered. Antibody beads (50 μL) were added to each well, filtered and washed once with 150 μL of washing buffer. Twenty-five microlitres of serum standard buffer was added to all the sample wells, and later equal volume (25 μL) of serum was added to each well, incubated at room temperature for 60 min. Later, washing was done three times, after which 25 μL premixed detection antibody was added, incubated for 30 min on the shaker at room temperature. After, washing and filtration was done three times. During each wash 150 μL of washing buffer was used. Streptavidin-PE (50 μL) was later added and incubated for 30 min, later washed and filtered three times. After the third wash the plate was prepared for analysis. Reading buffer (120 μL) was added to each instrument.

### Measurement of transferrin

Serum transferrin was measured according to manufacturer’s instruction (ELISA kit from Alpha diagnostic International, USA, Catologue number 6390). Briefly, 100 uL of calibrators, control and samples were pipette into pre-designated wells already coated with anti-transferin antibodies, all in duplicates. This was incubated at room temperature (RT) for thirty minutes. The plate was covered during incubation. The contents of the wells were aspirated following incubation. Thereafter, each well was completely filled with diluting wash solution and aspirated. This was repeated three times, for a total of four washes. Thereafter, 100 uL of appropriately diluted enzyme-antibody conjugate was added to each well and incubated at room temperature for 30 min in the dark. The plate was covered and kept level during incubation. Washing was done and plates blotted four times as done previously. Thereafter, 100 μL of 3,3′,5,5′ tetramethylbenzidine (TMB) was added to each well and incubated for 10 min in the dark; after which 100 uL of stop solution was added to each well. The absorbance of each well was read at 450 nm.

### Statistical analysis

Data analysis was done using the GraphPad Prism Version 5.00 for Windows, GraphPad Software, San Diego California, USA, [21]. Data are expressed as the mean with standard deviation (SD) unless otherwise stated. Data were log transformed to ensure normal distribution before one-way analysis of variance (ANOVA, with Tukey’s post-test, two tailed), were performed. Pearson correlation analysis was performed on the transformed data of variables to compare the relationship between them. Values were considered significant when p < 0.05.

## Results

### High levels of anti-EPO antibody in the semi-immune mice strains

**Figure 2:**
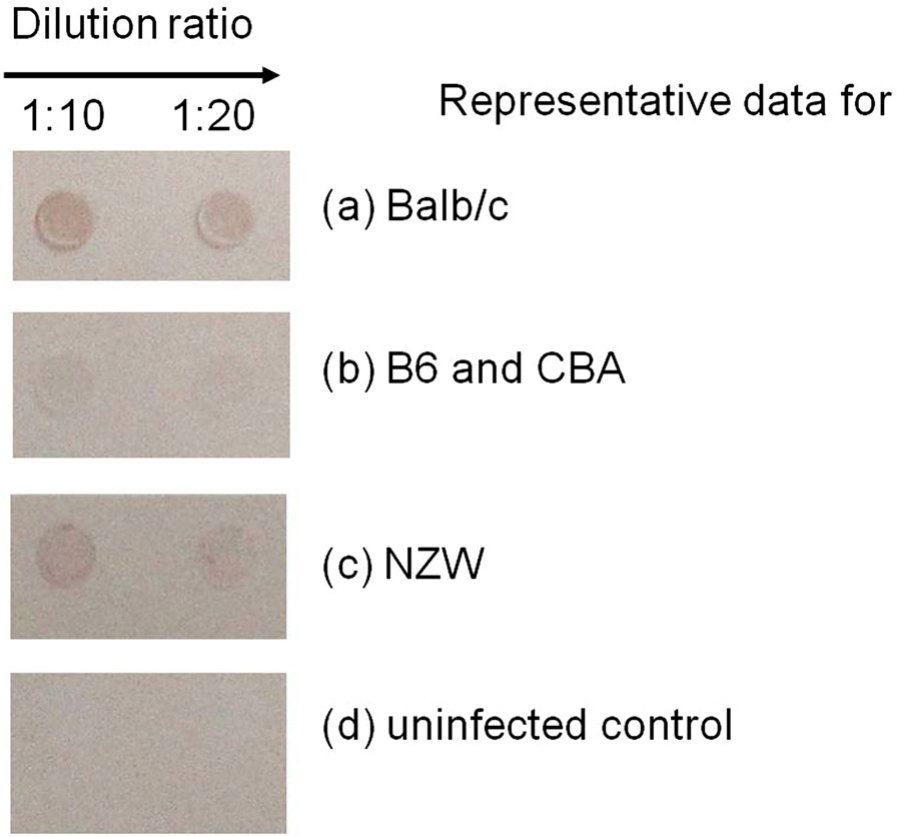
**Detection of Anti-EPO antibody in sera of different semi-immune mice strains and control at different dilution.** Sera was taken from blood harvested at minimum Hb (i.e. maximum Hb reduction) and screened for anti-EPO antibody by immunodot blot. The above data are representative data for each semi-immune mice strain. Number of mice used is two per strain. Direction of arrows indicate extent of serum dilution by a factor of 2. Upper panel **(a)** shows immuno dot blot for Balb/c; panel **(b)** immediately below (that of Balb/c) is the immuno dot blot for B6 and CBA while the third lowest panel **(c)** is the immuno dot blot for NZW and lower panel **(d)** is the immuno dot blot for uninfected control mice.

**Figure 3:**
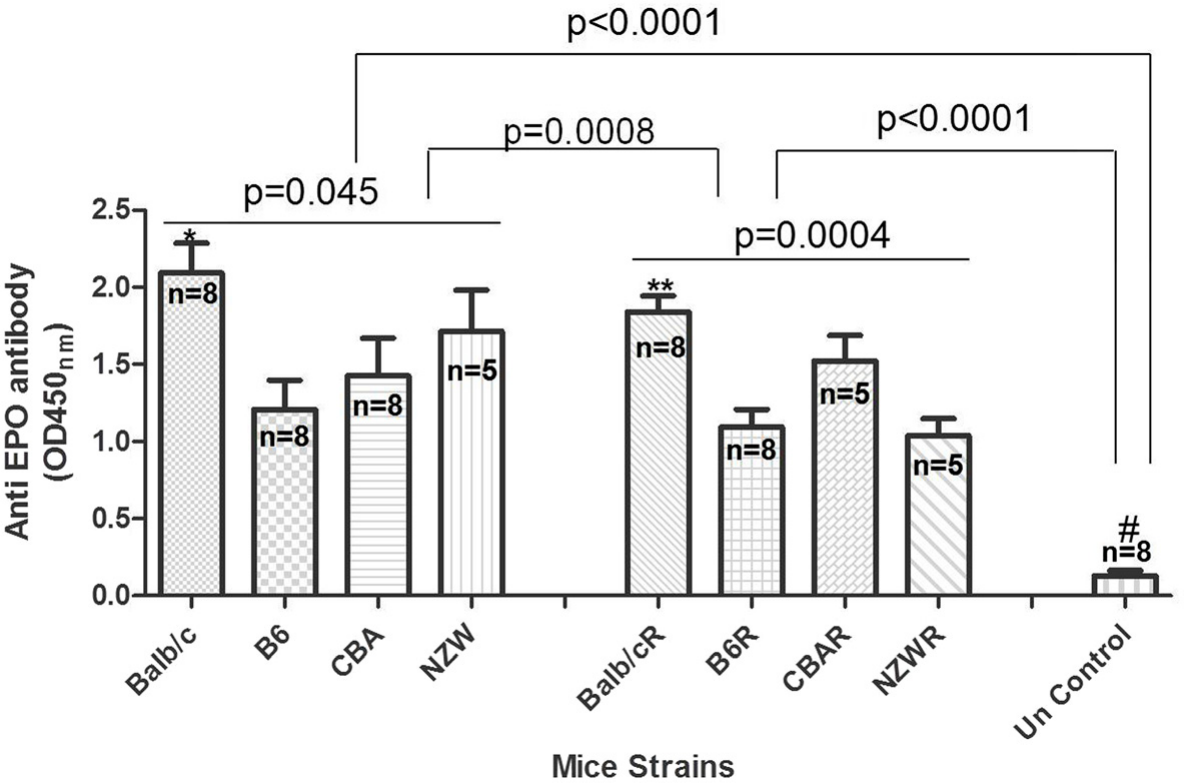
**Levels of anti-EPO antibody in the semi-immune mice at infection and recovery.** These are anti-EPO antibody level at infection and recovered (R). Blood was harvested for serum at minimum Hb (i.e. maximum Hb reduction, at infection) and recovery (when parasitaemia is cleared). Means of Anti-EPO Ab at infection and recovery differed, p = 0.045 and 0.0004 respectively. *Balb/c values significantly higher when compared with B6 (p < 0.05). **Balb/c values at recovery different from B6 and NZW (p < 0.05). ^#^Significantly lower when compared with the others at infection and recovery (p < 0.0001). Values at infection and recovered differed (p = 0.0008). Recovery refers to time at which mice has been rested for two weeks after treatment with chloroquine/pyrimethamine for 6 days or until parasitaemia is zero. Values are means and errors bars being standard deviation (SD). Data was analysed by ANOVA after they were log_10_ transformed. n refers to number of samples. These are pooled data of two separate experiments. Un Control means uninfected control (consist of 2 mice per strain, giving a total of 8 mice).

Sera of the mice strains were screened by immunodot blot for the possible identification of anti-EPO antibody levels. It can be seen from Figure 2 that sera from all the mice strains showed the presence of anti-EPO antibody, but negative for uninfected control sera, suggesting that parasitaemia might contribute to the production of the anti-EPO antibody. The anti-EPO antibody levels were measured, at infection and at recovery. Anti-EPO antibody was significantly different in the mice strains (mean OD_450nm_ values: Balb/c (2.1); B6 (1.3); CBA (1.4); NZW (1.7)) at infection (p = 0.045). While Balb/c anti-EPO Ab levels at infection differed from B6 (p < 0.05), the others were not significantly different when paired. The anti-EPO antibody is significantly higher in all four mice strains at infection when compared with the uninfected control (mean OD_450nm_ value = 0.1) (p < 0.0001). Anti-EPO Ab levels at recovery in the mice strains (Balb/c, 1.8; B6, 1.1; CBA, 1.5; NZW, 1.0) were significantly different, p = 0.0004. Again anti-EPO Ab levels of Balb/c at recovery were significantly different from that of B6 and NZW (p < 0.05), while the others did not differ (p > 0.05). The means of anti-EPO Ab levels at infection and recovery differed (p = 0.0008) (Figure 3).

### Association of anti-EPO antibody with Hb loss

**Figure 4:**
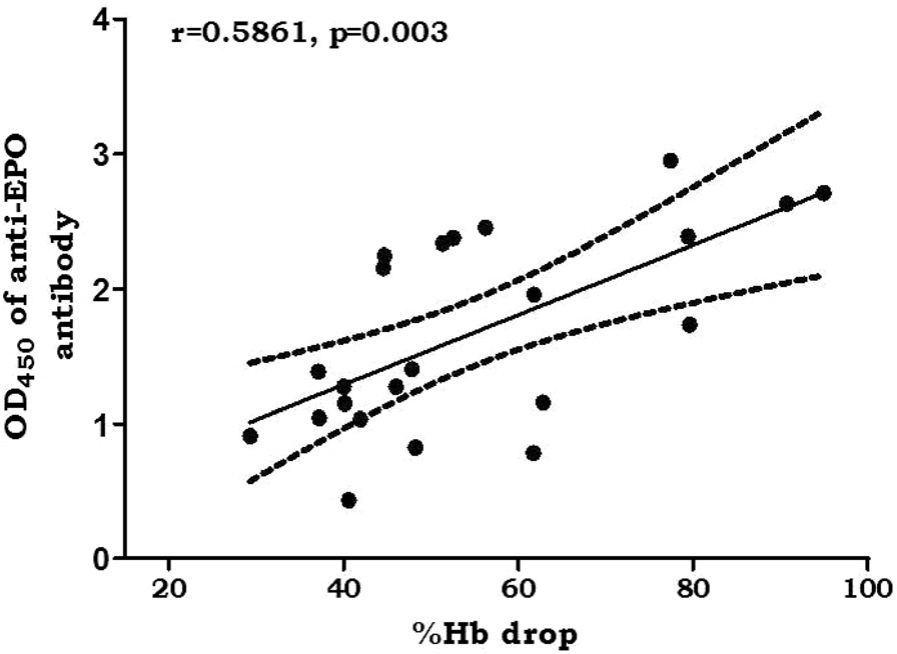
**Correlation between %Hb loss and anti-EPO antibody (all mice combined).** Total number of samples n = 23 (Balb/c (8), B6 (5), CBA (6), NZW (4)). Dotted lines are 95% confidence intervals and the continuous line is the line of best fit. r is Pearson coefficient. Blood was harvested at minimum Hb in the course of monitoring the haematological and parasitological parameters. These are values of two separate experiments.

**Figure 5:**
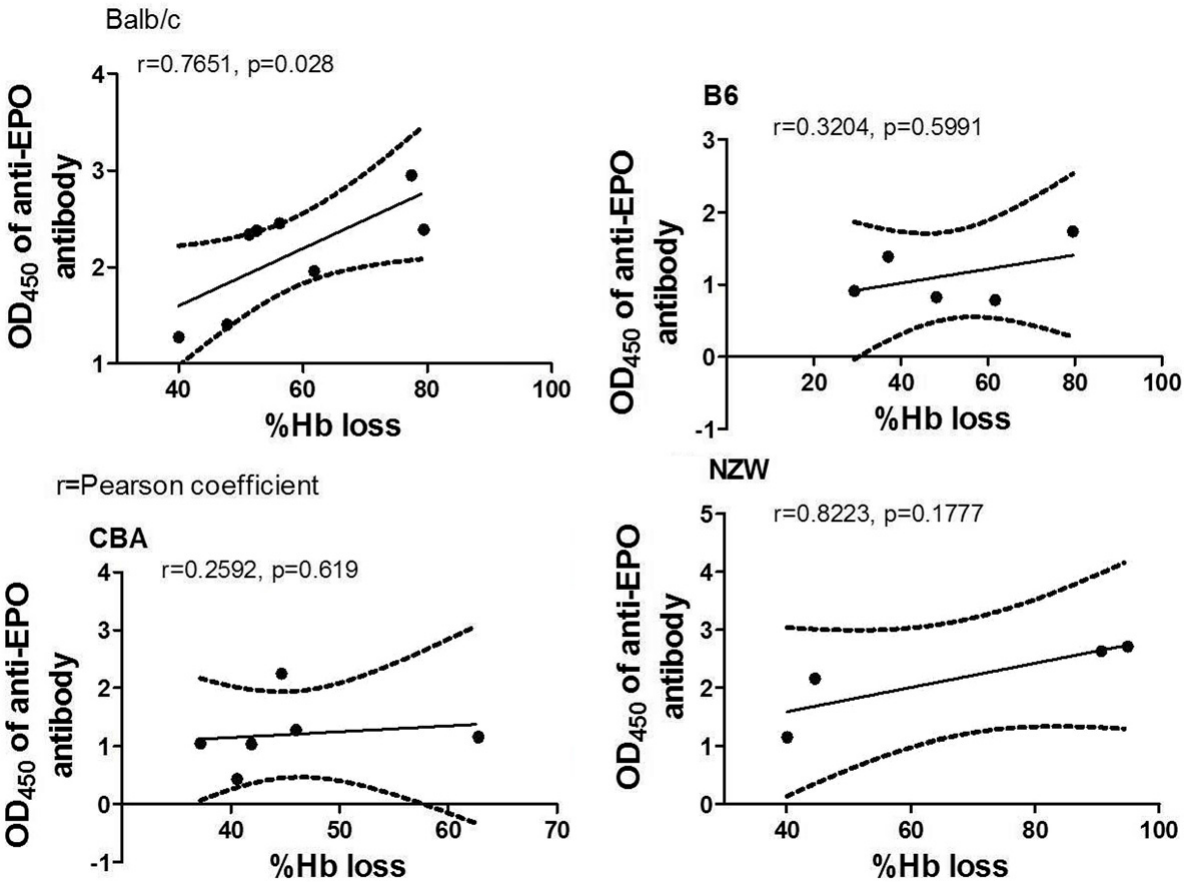
**Correlation between %Hb loss and anti-EPO antibody (per mice strains).** Total number of samples n = 23 (Balb/c (8), B6 (5), CBA (6), NZW (4)). Dotted lines are 95% confidence intervals and the continuous line is the line of best fit. r is Pearson coefficient. Blood was harvested at minimum Hb in the course of monitoring the haematological and parasitological parameters. These are values of two separate experiments.

To explore the association of anti-EPO antibody with anaemia, the level of anti-EPO antibody was plotted against Hb loss in all the mice strains giving interesting results as seen in Figure 4 (combined results for all the mice strains) and Figure 5 (individually). Anti-EPO antibody correlated significantly with Hb loss when all the mice strains was combined, Figure 4 (r = 0.5681; p = 0.003). Plotting anti-EPO antibody against Hb loss per mice strains (Figure 5), it was observed that the correlation was significant (r = 0.7651; p = 0.028) in only Balb/c but not in other strains B6 (r = 0.304; p = 0.5991), CBA (r = 0.2592; p = 0.619) and NZW (r = 0.8223; p = 0.1777).

### EPO level and its association with anti-EPO antibody

**Figure 6:**
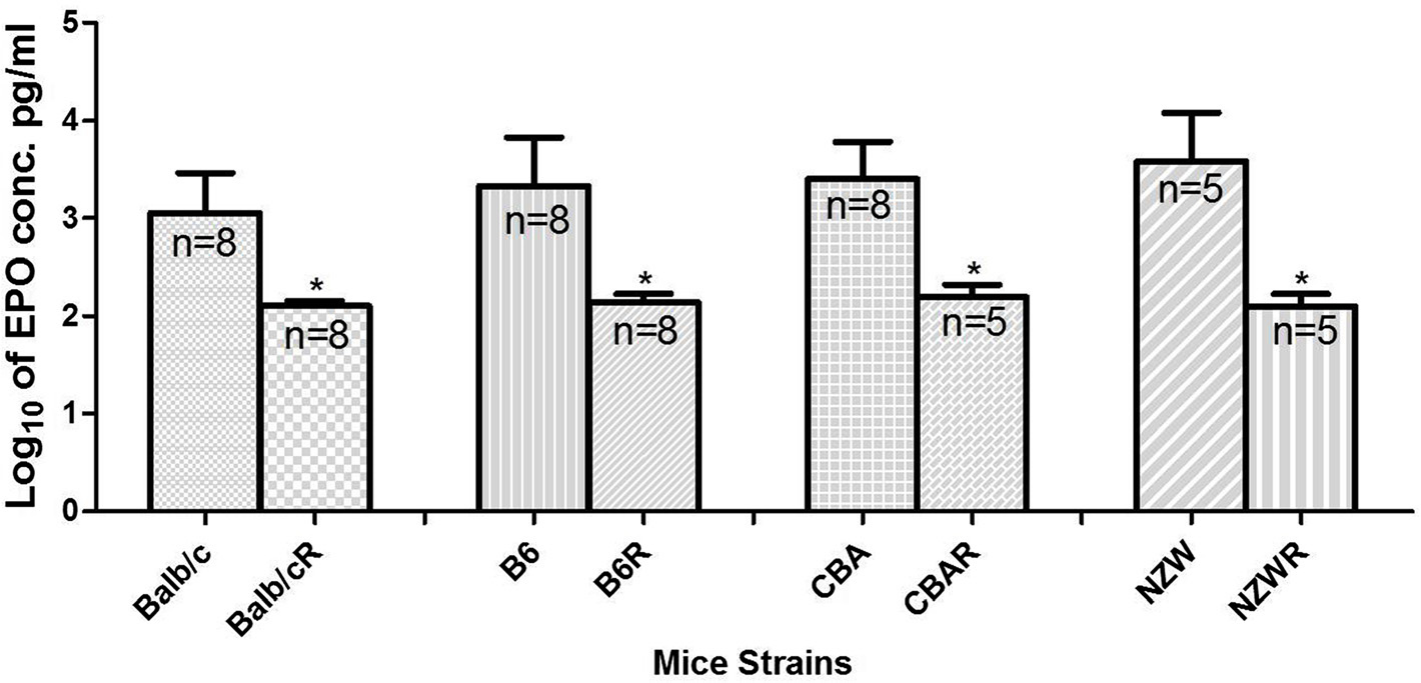
**EPO levels in the semi-immune mice strains at infection and recovery.** Blood was harvested for serum at minimum Hb at infection and at recovery. *Values at recovery are significantly lower than at infection for all the mice strains (p < 0.01). Values at recovery were not significantly different for all the mice strains. Recovery refers to time at which mice has been rested for two weeks after treatment with chloroquine/pyrimethamine for 6 days or until parasitaemia is zero. OD values from ELISA were converted to EPO concentration of pg/ml before it was transformed to log_10_. Values are means and errors bars being standard deviation (SD). Data was analysed by ANOVA after they were log transformed, with Tukey’s post test. n refers to number of samples. These are pooled data of two separate experiments.

To further understand the erythropoietic response in all the semi-immune mice strains the EPO levels were evaluated at both infection and at recovery. It was consistently observed for each mice strain that, EPO at recovery was significantly lower than that at infection (Figure 6), confirming earlier reports [5-7]. However, when EPO levels at infection was compared in all the mice strains, EPO levels in NZW was significantly higher than Balb/c (p < 0.05).

**Figure 7:**
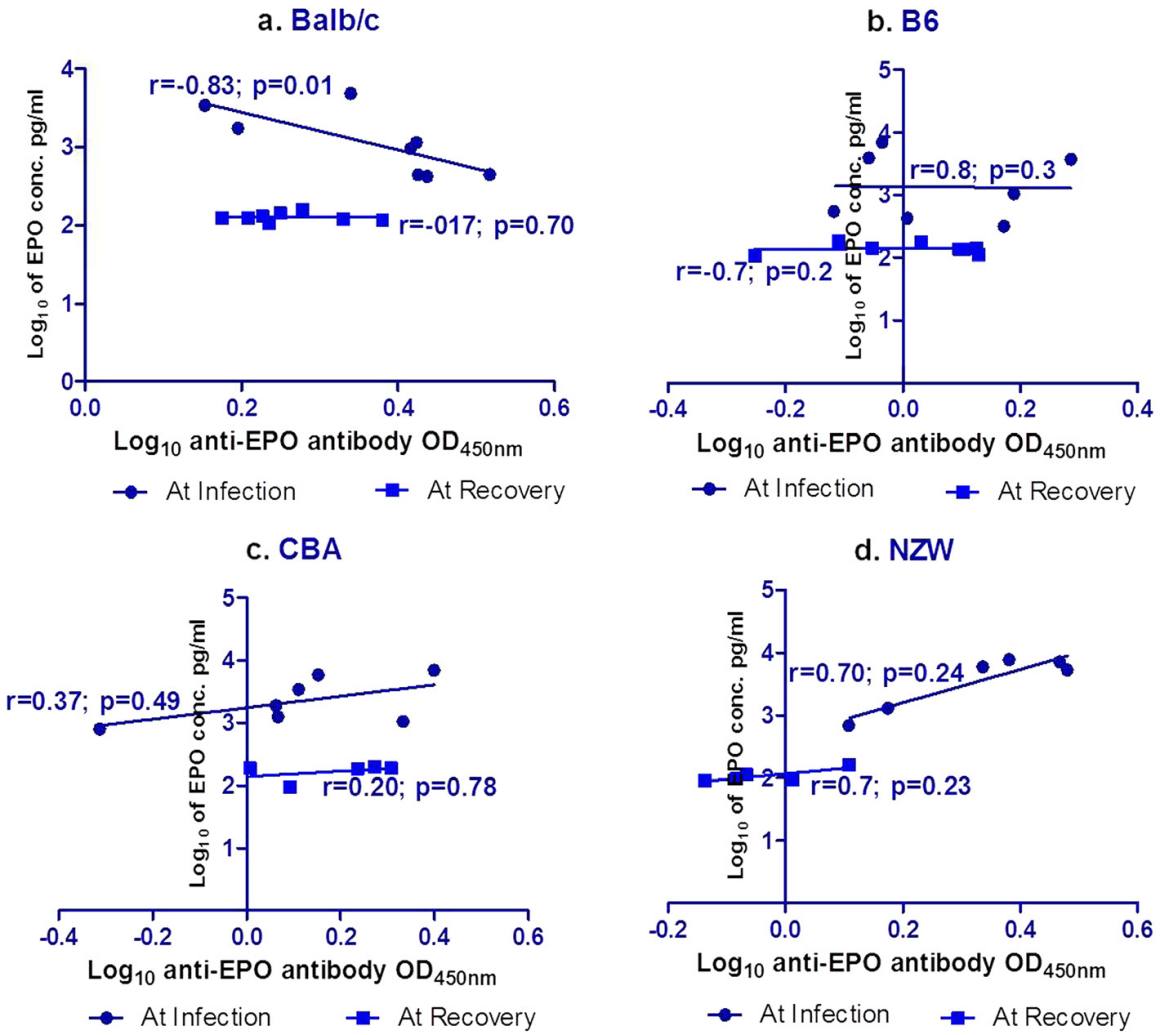
**Correlation between EPO and anti-EPO antibody at infection and recovery of all the semi-immune mice strains.** Blood was harvested for serum at minimum Hb at infection and at recovery for EPO and anti-EPO antibody measurement as indicated in materials and methods. Number (n) of mice per strain at infection, Balb/c (n = 8), B6 (n = 7), CBA (n = 7) and NZW, (n = 6); and at recovery, Balb/c (n = 8), B6 (n = 8), CBA (n = 5) and NZW (n = 5)). Graph **a**. upper left is correlation for Balb/c; graph **b**. upper right is correlation for B6; graph **c**. lower left is correlation for CBA; graph **d**. is correlation for NZW. Continues line is the line of best fit, while r is Pearson coefficient. These are pooled data of two experiments. Log_10_ transformation of EPO was done on the EPO concentration of pg/ml after being converted from OD values from the ELISA readings. Anti-EPO antibody values (OD values) were also log_10_ transformed.

Since EPO level is a measure of how active the individual is able to respond to Hb drop, the association of EPO level with the anti-EPO antibody level in all the semi-immune mice strains was analyzed but found no significant correlation. However, when this association was analysed per mice strain, a significant negative association was found only in Balb/c mice strain at infection stage, Figure 7 (r = −0.83; p = 0.01). No significant association was found in the other mice strains both at infection and recovery, Figure 7.

### Cytokine levels and their relationship with anti-EPO antibody in the semi-immune mice strains

While IL-6 and INF-γ are known to suppress erythropoiesis, TNF and IL-10 are thought to contribute to the degree of anaemia in children with falciparum malaria [22,23]. These cytokines were measured to evaluate the extent to which they may be implicated in anaemia and anti-EPO antibody production indirectly. IL-17 is thought to be associated with autoimmunity. Significantly high levels of IL6, IL-17 and INF-γ were observed in Balb/c, p < 0.05 (Table 1). Interleukin 10 (IL-10) and TNF were similar in all the mice strains, p = 0.08, 0.07 respectively; TNF/IL10 ratio on the other hand were significantly different in all the mice strains, p = 0.02. To further understand the relationship between the cytokines and anti-EPO antibody, anti-EPO antibody was plotted against the cytokine levels. It was observed that there was no correlation (p > 0.05) between all the cytokines and anti-EPO antibody in all the mice strains.

**Table 1:** Cytokine levels in the semi-immune mice strains and uninfected controls

**Cytokine (pg/ml)**	**Mouse strains (number)**					P value*
	**Balb/c (8)**	**B6 (4)**	**CBA (6)**	**NZW (4)**	**Uninfected control (4)**	
IL6 (SD)	6692.0	513.1^**a**^	136.7^**b**^	464.1^**d**^	71.60^**c**^	0.0001
	(4443.0)	(757.9)	(76.8)	(664.6)	(30.63)	
IL17 (SD)	6052.0	205.3^**a**^	4.59^**b**^	89.7^**d**^	4.05^**c**^	< 0.0001
	(4202.0)	(323.0)	(0.71)	(121.0)	(0.5)	
IL10 (SD)	13.97	12.51	23.14	16.31	4.625	0.08
	(12.67)	(5.9)	(33.3)	(10.15)	(1.33)	
INFγ (SD)	6785.0	715.7	93.74^**b**^	413.9^**d**^	6.5^**c**^	0.001
	(4805.0)	(1218.0)	(83.7)	(799.8)	(0.38)	
TNF (SD)	54.86	202.5	16.24	18.65	16.15	0.07
	(35.4)	(410.4)	(1.08)	(3.835)	(3.85)	
TNF:IL10 (SD)	4.5	1.7	1.5	1.5	4.0	0.002
	(1.0)^**#**^	(0.6)	(0.7)	(0.9)	(2.4)	

Blood was harvested for serum at minimum Hb. Values are pooled data of two separate experiments. *P values done by ANOVA was performed on the log_10_ transformed data. Analysis was done by ANOVA with Tukey’s post test. ^a, b, c, d^ Statistically lower than Balb/c. ^#^Significantly higher than the other strains.

### Transferrin levels in the semi-immune mice strains

**Figure 8:**
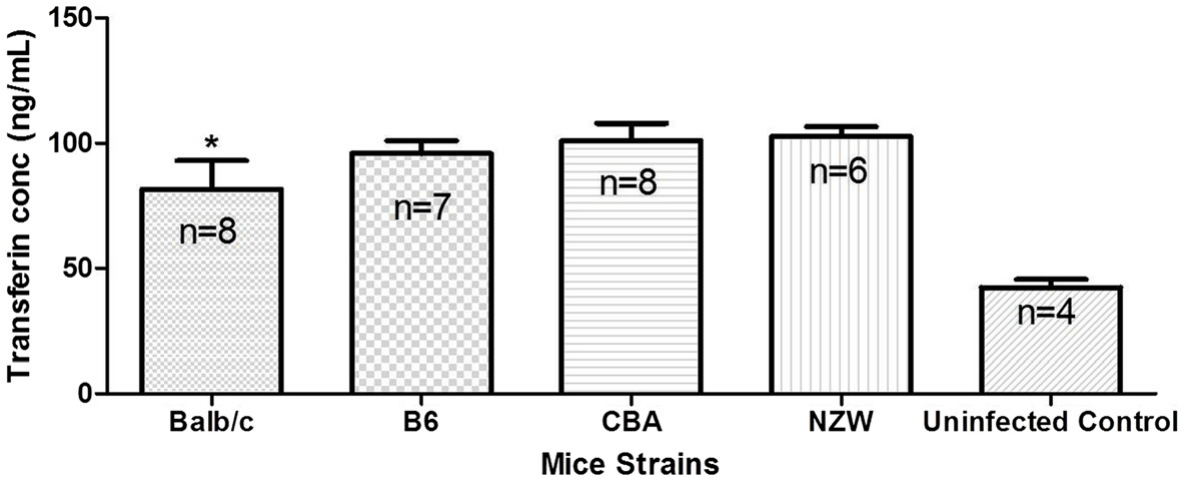
**Transferrin levels in the semi-immune mice strains.** Blood was harvested for serum at minimum Hb. *Significantly lower than all the mice strains and significantly higher than uninfected control (p < 0.0001). B6, CBA and NZW are not significantly different but significantly higher than uninfected control. Values were log_10_ transformed and analysed with ANOVA. These are pooled data from two separate experiments.

Transferrin is known to regulate iron metabolism and also an indicator of inflammatory response during infection. Thus, to evaluate the extent to which iron is metabolized at minimum Hb (maximum Hb reduction, see Figure 1 in [2]) and possible implication with immune response transferrin levels were measured at infection and found out that the levels was significantly low in the Balb/c when compared with the other mice strains. An elevated level of transferrin was also observed at infection for all the semi-immune mice strains when compared with the uninfected mice (Figure 8).

## Discussion

To the knowledge of the authors this is the first report evaluating the levels of endogenous anti-EPO antibodies during malarial infection. The study describes a correlation between anti-EPO antibody with Hb loss. The data presented here show that anti-EPO antibody is produced during malaria infection. It is also observed in this study that host genetic factors are playing a role in the extent of anti-EPO antibody production during anaemic condition.

The positive correlation of anti-EPO antibody with %Hb loss (in Balb/c) and EPO levels in this study has been observed in another study of auto-immune condition [9]. This study, however, do not have enough data to suggest a neutralizing effect anti-EPO antibody has on EPO during Hb loss, to keep a certain minimum amount of RBC in the pool until parasite is cleared. Further studies to evaluate that will be very informative. It is interesting to note that Balb/c which has relatively low parasitaemia, high survival rate [2], has a significant positive correlation of anti-EPO antibody with %Hb loss and EPO levels. The low EPO level observed in this study, indicative of low iron level; and a relationship with protection has been observed in a recent field data in humans [24]. The explanation is that during low iron level an unfavourable internal environment is created which prevents the parasites from full proliferation. This agrees with nutritional immunity from a study on bacterial infections where protection for individuals with iron deficiency on disease severity was observed [25].

In this study, no significant correlation between anti-EPO antibody and the cytokine levels in all the mice strains was observed. However, it is tempting to indicate that the significantly high levels of IL-6, IL-17 and INF-γ observed in Balb/c, (Table 1), may signify synergistic effect between them and anti-EPO antibody resulting in low parasitaemia hence high survival, due to low iron level. It is not clear why similar trend of inflammatory cytokine was not observed in CBA and B6 even though similar parasitaemia was observed in them and Balb/c. Low-density parasitaemia and its treatment have been found to induce a mild increase in IL-6 and serum hepcidin concentrations [26]. Thus the high level of IL-6 may impair bioavailability of iron resulting in low iron level leading to low parasitaemia. High levels of these two cytokines (IL-6 and INF-γ) in Balb/c suggest suppression of erythropoiesis but the mechanism(s), however, remain to be clarified. This findings support other studies where IL-6 and INF-γ were postulated to suppress erythropoiesis [27]. Even though TNF and IL-10 are thought to contribute to the degree of anaemia in children with falciparum malaria, no significant difference was observed among the mice strains in this study, and even when compared with uninfected control. IL-17 promotes inflammation [28] by activating cells to produce more pro-inflammatory cytokines and also thought to be associated with autoimmunity [29] suggesting that these phenomena may be associated more with Balb/c.

The hall mark of malaria in acute cases is anaemia. This type of anaemia occurs within a very a short period and sudden. However, this study mirrors chronic infection as is the case of individuals in malaria endemic areas where persistent low Hb is observed for a relatively long period of time. Many chronic infections such as malaria are characterized by a generalized state of chronic inflammation. Furthermore, low Hb level is associated with chronic infection thus low iron level. Another way of estimating iron level in the serum is via transferrin level. Although transferrin is involved in iron trafficking [30,31], it is also an indicator of inflammatory response [32]. Thus, transferrin being an acute phase reactant gets elevated in inflammatory response due to malaria infection. This study concurs with this observation where in all the mice strains; transferrin level was significantly higher in the infected and recovered semi-immune mice than the uninfected level (Figure 8). Considering the extent to which these inflammatory cytokines (IL-6 and INF-γ) could be interacting with anti-EPO antibody to lower iron level, resulting in relatively low parasitaemia as discussed above; it was surprising to observe significantly low level of transferrin in Balb/c. This is because a patient with an increased serum transferrin level often suffers from iron deficiency anaemia [33]. On the other hand a patient with decreased plasma transferrin can suffer from iron overload diseases and protein malnutrition. This shows that transferrin imbalance can have serious health effects for those with low or high serum transferrin levels. Balb/c having the least level of transferrin also suggests that they (Balb/c) had the most inflammatory process, which is backed by relatively high levels of IL-6 and INF-γ in Balb/c than the other mice strains (Table 1). Additionally, clinical significance of low transferrin level may be indicative of liver damage as a result of liver iron overdose. Also elevated levels of transferrin in the other mice strains are indicative of enhanced erythropoiesis and relatively lower inflammatory response as compared to Balb/c.

In conclusion, the data presented here show that *Plasmodium* infection influences anti-EPO production in all mice strains; and correlates more with Hb loss in one mice strain suggesting host factors is involved. Further studies to evaluate the kinetics of the anti-EPO antibody and its neutralization of EPO activity will help deduce and understand further the development of anaemia in malaria.

## Competing interest

The authors declare that they have no competing interests.

## Authors’ contributions

NTH and KH designed the work with GKH. TY, MNS and MSC carried out animal experiment and Immunodot Assay with GKH. GKH, MNS, MK, and MSC designed and carried out the ELISA. GKH drafted the manuscript with MNS, NTH and KH, who were also involved with data analysis as well extensive revision of the manuscript for intellectual content. All authors read and approved the final manuscript.
